# Interaction of the synthetic antithrombotic peptide P10 with thrombin: a spectroscopy study

**DOI:** 10.1039/c9ra02994j

**Published:** 2019-06-11

**Authors:** Fangyuan Chen, Han Jiang, Wenwei Chen, Guangrong Huang

**Affiliations:** Key Lab of Marine Food Quality and Hazard Controlling Technology of Zhejiang Province, College of Life Sciences, China Jiliang University Hangzhou China grhuang@126.com +86 571 8687 5628

## Abstract

Thrombin is a critical serine protease in the coagulation system and is widely used as a target protein for antithrombotics. Spectroscopic analysis is a simple and effective method that is used to study the interaction between small molecules and proteins. In this study, the interactions of a potential antithrombotic peptide AGFAGDDAPR (P10) with thrombin were investigated by fluorescence spectroscopy, UV-vis spectroscopy, circular dichroism, Fourier-transform infrared spectroscopy and Raman spectroscopy, respectively. The results showed that the peptide P10 bonded to thrombin *via* hydrogen bonding and van der Waals forces, resulting in fluorescence quenching. And, the secondary structure of thrombin changed, the β-sheet decreased, and the random coil increased. The peptide P10 bonded to proline and lysine, and changed the space structure of thrombin, resulting in inhibition of thrombin activity. The results contributed to exploration of the mechanism of this potential antithrombotic drug interaction with thrombin in order to provide a preliminary understanding of the pharmacodynamic properties of P10.

## Abstract 2

Thrombin is a critical serine protease in the coagulation system and is widely used as a target protein for antithrombotics.

## Graphical abstract

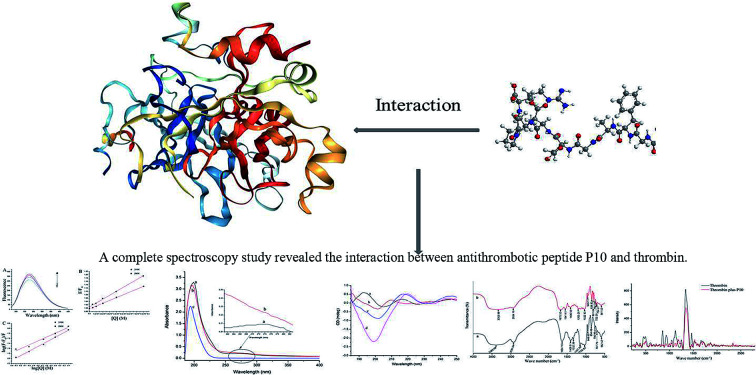

## Introduction

The coagulation process is a cascade of proteolytic reactions, divided into an intrinsic pathway and an extrinsic pathway. Although the cascade starts with two different mechanisms, its ultimate goal is to convert soluble fibrinogen to insoluble fibrin. Both pathways are combined at the activation step of thrombin in the cascade.^1^ Therefore, thrombin is considered as a critical serine protease in the blood coagulation process.^2^ In general, thrombin consists of a 36-residue light chain and a 259-residue heavy chain that can be mainly divided into the active site, exosite 1 and exosite 2, which are the regions where thrombin exerts its coagulation function.^3,4^ The thrombin active site cutting fibrinogen into fibrin is located in the gap formed at the interface of the two barrels.^5^ The substrate recognition of thrombin is regulated by two external sites responsible for substrate turning and orientation in the protease catalytic site. Two exosites regulate the substrate recognition of thrombin, which are remote from the active site and responsible for substrate turning and orientation.^6^ Exosite 1 binds to the extracellular domain of fibrinogen, thrombomodulin and the type-1 protease-activated receptor on the platelet. Exosite 2 binds to β2-glycoprotein-1 and determines the position of thrombin on the platelets by interacting with glycoprotein Ibα receptors.^7–9^

Peptides have attracted great interest in medical or health protection in the past few decades. Various peptides have been isolated and identified from natural materials and have demonstrated many biologically active functions such as bacteriostatic, antioxidant, hypotensive, hypoglycemic, *etc.*^10,11^ Many naturally occurring antithrombotic biologically active peptides have been found to be specific inhibitors of thrombin. Typically, a 60-amino acid peptide purified from the extract of *Ornithodoros moubata*,^12^ a peptide called draculin isolated from the saliva of the *Desmodus rotundus*^13^ and AduNAP4 from the human hookworm^14^ have already displayed antithrombotic activity *via* interacting with thrombin. In particular, hirudin, a peptide with 65 or 66 amino acids extracted from leeches, has been widely studied as a highly effective direct thrombin inhibitor.^6,15,16^ Some peptides derived from organism proteins by enzymatic hydrolysis also exhibit antithrombotic activity, such as peptide FQSEEQQQTEDELQDK from casein hydrolysate.^17^ In particular, a peptide P10 (AGFAGDDAPR) isolated from *Tenebrio molitor* with enzymatic hydrolysate exhibited antithrombotic activity in our previous study.

Information about the binding between antithrombotic peptides and thrombin is the basis for understanding the pharmacodynamic properties of antithrombotic peptides. At present, studies on the interaction of antithrombotic peptides and thrombin mainly focus on methods of X-ray diffraction and molecular simulation.^18,19^ But they have limitations in the understanding of molecular structure, function, and physical properties in solution which is required by the latest developments in protein structure research. Spectral methods that are not limited to the morphology of molecules have attracted great interest and have been used to effectively reveal the structure of proteins in solution.^20^ In this work, the interaction between an antithrombotic peptide P10 and thrombin was studied by fluorescence spectroscopy and UV-vis absorption spectra. Circular dichroism (CD), Fourier-transform infrared spectroscopy (FT-IR) and Raman spectroscopy were used to analyze the effect of peptides on the thrombin structure. The mechanism of interaction of the peptide with the thrombin molecule is expected to be initially revealed. At the same time, it also aims to provide a comprehensive and detailed spectroscopic analysis method for revealing the interaction between small molecule ligands and macromolecular receptors.

## Results and discussion

### The antithrombotic activity of the synthesized peptide P10

**Fig. 1:**
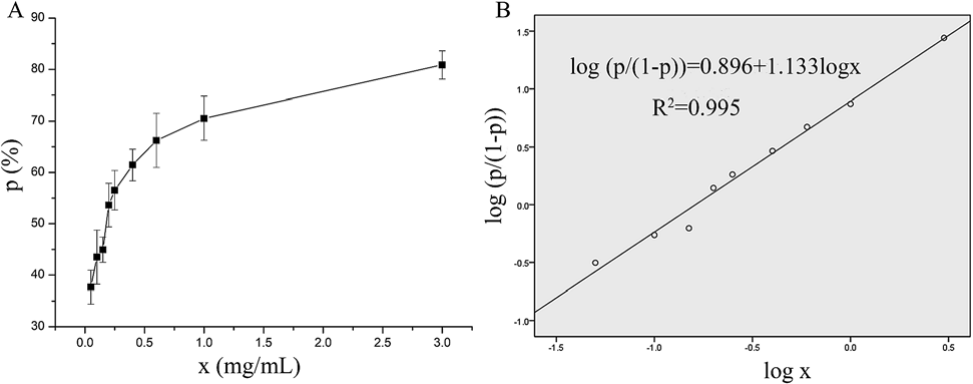
The antithrombotic activity of P10. (A) A visual map of the relationship between P10 concentration and the inhibition rate of the thrombin. (B) The probit model for the logarithm of the inhibition rate of the thrombin and the P10 concentration. The *x* represents the concentration of P10, and *p* represents the inhibition rate of the thrombin (antithrombotic activity).

The *in vitro* antithrombotic activity of P10 was measured quantitatively and the results are shown in Fig. 1A. The peptide concentration showed a dose-responsive behavior to the antithrombotic activity. These data were processed using the probit program in SPSS to give the IC_50_ = 0.16 mg mL^−1^ (inhibition of 50% thrombus formation) (Fig. 1B).^21^ Ren *et al.*^22^ purified an anticoagulant peptide with the sequence VEPVTVNPHE from *Buthus martensii* Karsch, which inhibited thrombin activity with an IC_50_ of 0.012 mg mL^−1^. In addition, three peptides SWAQL, GNHEAGE and CFNEYE were purified from the Alcalase 2.4L hydrolysate of peanut protein, showing complete inhibition of thrombin activity at a concentration of 0.4 mg mL^−1^.^23^ However, the synthesized KNAENELGEVTVR illustrated 67.21% antithrombotic activity at a concentration of 1 mg mL^−1^.^24^ Although P10 had a low antithrombotic activity, it may reduce the probability of bleeding or other side effects.^25^

### Fluorescence quenching of thrombin by P10

#### Fluorescence quenching

**Fig. 2:**
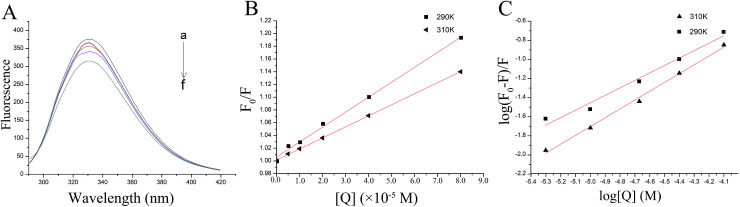
The fluorescence quenching of thrombin by P10. (A) The fluorescence spectra of 5 μM thrombin mixed with various concentrations of P10 (*λ*_ex_ = 280 nm) at 290 K. The concentrations of P10 from ‘‘a” to ‘‘f” were 0, 5 μM, 10 μM, 20 μM, 40 μM and 80 μM. (B) The Stern–Volmer plots for the fluorescence quenching of thrombin by P10 at 290 K and 310 K. (C) Plots of log (*F*_0_ − *F*)/*F* against log [Q] for the P10 quenching effect on thrombin fluorescence at 290 K and 310 K.

Fluorescence spectroscopy provides information on the structure and kinetics of the protein that is often used to study protein folding and binding reactions.^26^ The thrombin molecular structure contains 11 tyrosine residues, 11 phenylalanine residues and 8 tryptophan residues. The fluorescence intensity of tryptophan is greatest in these fluorescent chromogenic amino acids. When the excitation light is set to 280 nm, it can be considered that the fluorescence exhibited by the protein is derived from the tryptophan in the molecule.^27^ Like most proteins, conformational changes, subunit binding, substrate binding and denaturation can lead to changes in the intrinsic fluorescence of thrombin.^28^ The effect of P10 on the fluorescence intensity of thrombin is shown in Fig. 2A. The excitation wavelength was fixed at 280 nm, and thrombin had a strong fluorescence emission band at 331 nm. The fluorescence emission peaks at 331 nm indicated that the tryptophan residues were the major contributing fluorophores in all three protein species. Obviously, the fluorescence intensity of thrombin reduced gradually with the P10 concentration, but the maximum emission wavelength and shape of the peak was not affected. This suggests that P10 might interact with thrombin and quench its intrinsic fluorescence. No spectral shift of the emission spectrum was observed in the P10 plus thrombin mixture, indicating that the hydrophobicity and polarity of the chromophore did not change.^29^

#### The quenching mechanism

The Stern–Volmer plot at 290 K and 310 K was plotted to determine the quenching mechanism (static quenching or dynamic quenching), as shown in Fig. 2B. *F*_0_ and *F* represented the fluorescence intensity of thrombin in the absence and presence of P10, respectively. And [Q] represented the concentration of the quencher. In general, the stability and quenching constants of the compounds formed under the static quenching mechanism decrease with increasing temperature. The interaction under dynamic quenching increases the number of effective collisions and enhances energy transfer, thus increasing the quenching constant of the fluorescent material as the temperature increases.^26,30^ The ratio of *F*_0_/*F* was linear with the concentration of P10, and the slope of the quenching curve at 290 K was higher than that at 310 K (Fig. 2B).

This indicated that fluorescence quenching between thrombin and P10 was static quenching.

To confirm this conclusion, the fluorescence quenching data was further analyzed by the Stern–Volmer eqn (1).^31^ Where *K*_sv_ represented the quenching constant of the Stern–Volmer.1
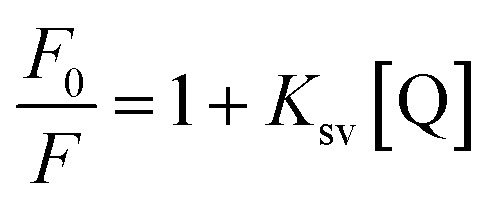


By calculation, the values of *K*_sv_ were 2.35×10^3^ M^−1^ (290 K) and 1.74×10^3^ M^−1^ (310 K). Generally, the *K*_sv_ value decreases with the temperature increases, indicating static quenching. On the contrary, it indicates dynamic quenching. The results showed that the quenching process of thrombin by P10 accorded with the static quenching regular pattern.

#### The binding constants and the number of binding sites

The binding constant (*K*_b_) and the number of binding sites (*n*) can be obtained from eqn (2) when small molecules are independently bound to a set of equivalent sites on the macromolecule:^33^2
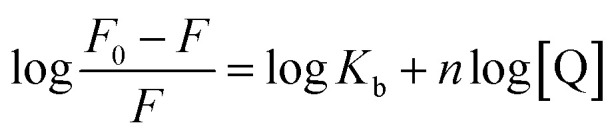


The plot of the P10 plus thrombin complex at 290 K and 310 K is shown in Fig. 2C, and the corresponding calculations are shown in Table 1. It was found that the *K*_b_ value decreased with increasing temperature, indicating that the stability of the P10 plus thrombin complex decreased with increasing temperature. However, the *K*_b_ value was always in the order of 10^2^, indicating that the binding of P10 to thrombin was not very strong. The weak interaction between P10 and thrombin could reduce the clotting activity of thrombin to a certain extent, and was more likely to reduce the possibility of major bleeding.^25^ And the value of *n* is approximately equal to 1, indicating that the thrombin and P10 interaction is 1 : 1.

**Table 1:** Binding constant *K*_b_ and the relative thermodynamic parameters of the system of P10 plus thrombin

*T* (K)	*K*_b_ (×10^2^ M^−1^)	*n*	Δ*H* (kJ mol^−1^)	Δ*S* (J mol^−1^ K^−1^)	Δ*G* (kJ mol^−1^)
290	2.35	0.779	−43.90	−63.87	−25.38
310	1.74	0.924	−43.90	−63.87	−24.10

#### Thermodynamic parameters

Thermodynamic parameters: free energy (*G*), standard enthalpy (*H*) and standard entropy (*S*) provide insight into the binding mode (including possible reactions and forces). When the temperature changes within a small range, *H* can be considered to be a constant. The thermodynamic parameters are evaluated using eqn (3) and (4), where *R* is the gas constant (8.31 K^−1^ mol^−1^ J) and *T* is the Kelvin temperature:^26^3
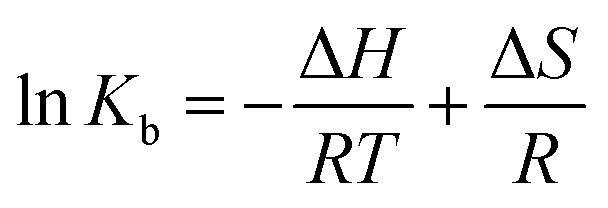

4 Δ*G* = Δ*H* − *T*Δ*S* = −*RT* ln *K*_b_

The values for the thermodynamic parameters interaction of P10 with thrombin are shown in Table 1. The changes in Gibb free energy (Δ*G*) < 0 indicated that the binding process of the interaction between P10 and thrombin was spontaneous. From a negative value of Δ*H* it can be inferred that the interaction between P10 and thrombin is an exothermic process, that is, an increase in temperature adversely affects the interaction. And Δ*S* was a negative value which is not favorable for the interaction of P10 with thrombin. Moreover, the bonding process was mainly due to hydrogen bonding and van der Waals forces.^34^

### UV-vis spectroscopy study

**Fig. 3:**
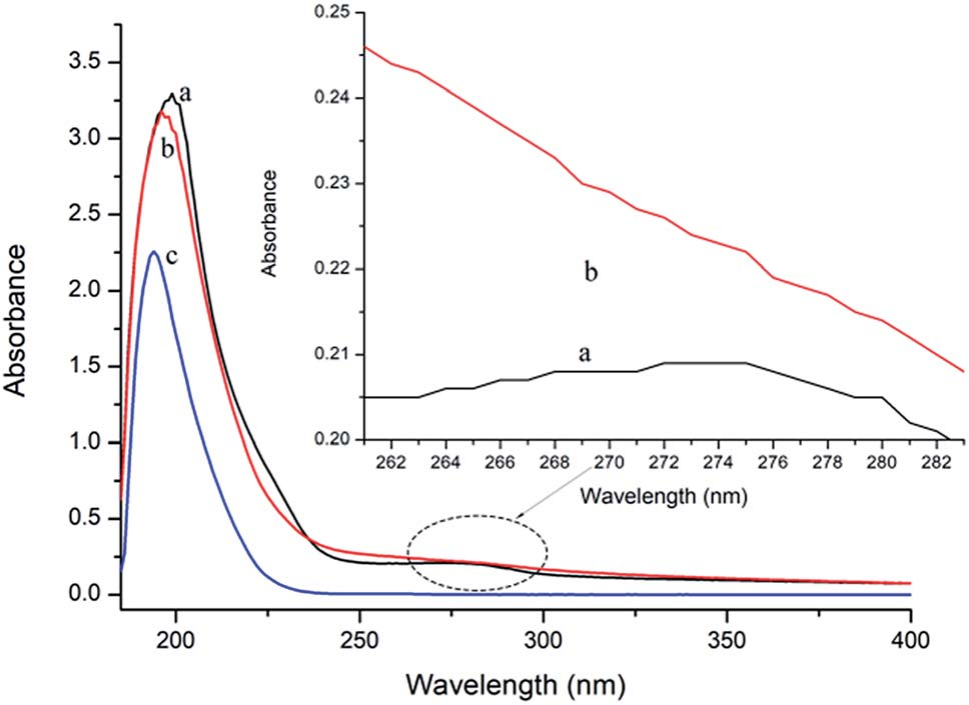
UV spectra of thrombin (a), thrombin plus P10 (b) and P10 (c). The concentration of thrombin and P10 was 5 μM and 80 μM, respectively.

UV-vis absorption measurement is a simple but efficacious method to explore the structural changes of proteins and to investigate protein–ligand complex formation.^26^ In this study, the spectra of P10 and thrombin in the presence and absence of P10 were obtained between 185 nm to 400 nm (Fig. 3). It can be seen that thrombin has two absorption peaks at 199 nm and 275 nm, respectively. By adding P10 to the thrombin solution, the peak intensities at 199 nm decreased with a slight blue shift (3 nm). And the peak at 275 nm basically disappeared.

In general, an absorption peak around 200 nm is caused by the π–π* transition of the C=O group on the amide bond.^35^ The interaction of P10 with thrombin caused the peptide chain of thrombin to shrink, resulting in absorbance around 200 nm reduction. And the addition of P10 to thrombin increases the polarity around the amide bond, resulting in an increase in the π–π* transition energy and a blue shift in the absorption peak. The UV absorption of the protein near the wavelength of 275 nm is mainly caused by the π–π* transition of tryptophan, tyrosine and the phenylalanine aromatic heterocycle.^36,37^ P10 tightened the thrombin structure to mask the hydrophobic groups of these aromatic amino acids, resulting in a weakened absorption peak. Dynamic quenching is mainly caused by the energy transfer of the receptor to the ligand by collision, and has no effect on the UV-visible spectrum of thrombin. In contrast, in static quenching, the UV-visible spectrum of the protein changes due to the complexation between the protein and the substance.^32^ Thus, the change in the UV-visible spectrum showed that P10 and thrombin formed a complex and further demonstrated that the main mechanism of quenching was static quenching.

### CD spectroscopy study

CD spectroscopy can measure the secondary structure of proteins in solution, such as α-helix, β-sheet, β-turn, and random coil. The far-ultraviolet region of the CD spectrum in the 178–250 nm wavelength range is the absorption peak range of the peptide bond, reflecting the main chain conformation.^38^ In general, when a ligand binds to a protein, it will cause a certain change in the secondary structure of the protein. Thus, in CD spectroscopy, conformational changes in the protein caused by the ligand can be assessed by measuring the protein secondary structure.^39^

**Fig. 4:**
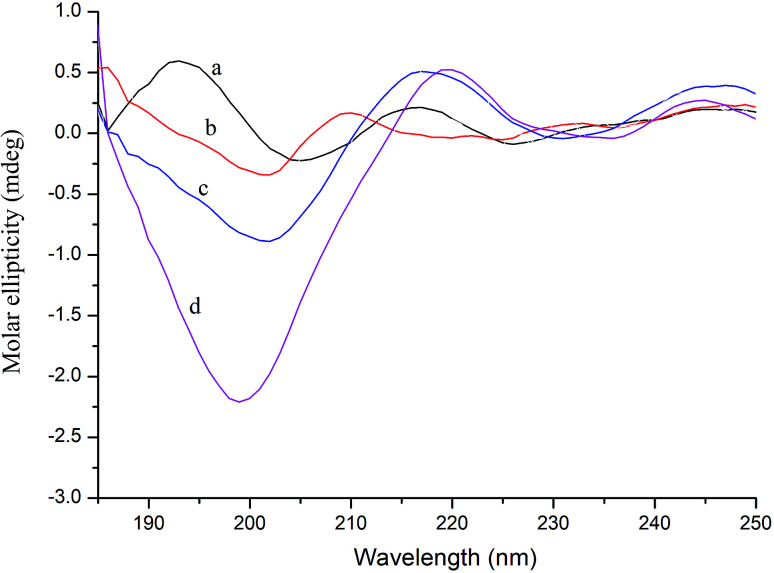
CD spectra of thrombin (a), thrombin plus 10 μM P10 (b), thrombin plus 40 μM P10 (c) and thrombin plus 80 μM P10 (d). All of the concentrations of thrombin were 5 μM.

As shown in Fig. 4, further evidence of conformational changes in thrombin was obtained by CD spectroscopy at different concentrations of P10 (0, 10 μM, 40 μM and 80 μM). The typical α-helix structure (two negative bands centered at 208 nm and 222 nm and positive band centers near 190 nm) was not found in this figure and the significant negative peak was at 206 nm.^32^ This was similar to the γ-thrombin structure described previously which was an interesting model for studying the conformation of thrombin activity.^40^ Although γ-thrombin has only 0.1% of α-thrombin activity, it can form a clot at high enzyme concentrations. Moreover, the interaction of the ligand with γ-thrombin also reflects the interaction of the ligand with α-thrombin to some extent.^41^ And the spectra showed that P10 resulted in an increase in the intensity of the negative peak of thrombin at 206 nm with a slight shift to longer wavelengths. The ratio of each secondary structure was calculated by the Yang *et al.* method^42^ in Table 2. The main secondary structure in this thrombin was β-sheet, accounting for 82.9%. As the amount of P10 increased, the β-sheet in the enzyme decreased and the random coil content increased. This suggested that the interaction of P10 with thrombin resulted in a change in the secondary structure of thrombin.

**Table 2:** Contents of the secondary structure of thrombin treated with different concentrations of P10

Sample	α-Helix	β-Sheet	β-Turn	Random coil
Thrombin	0	82.9	0	17.1
Thrombin plus 10 μM P10	0	82.5	0	17.5
Thrombin plus 40 μM P10	0	81.7	0	18.3
Thrombin plus 80 μM P10	0	79.1	0	20.9

The natural conformation as well as the specific functional domains is important for the activity of the enzyme.^43^ Loop (β-turn and random coil) is a very flexible conformation. As the loop content increases, there is more chance of moving to the active center or the bonding site. Thus, these sites that are binding to the substrate are covered, resulting in the failure of the substrate to bind to the enzyme, and ultimately the enzyme is inactivated.^44^ P10 increases the structure of the loop structure by interacting with thrombin molecules and changes the conformation of the thrombin molecule. Therefore, from a characterization point of view, the clotting activity of thrombin decreases as the amount of P10 increases. However, with the amount of P10 added from 0 to 80 μM, the change in the secondary structure of thrombin from β-sheet to random coil was only 3.8%, indicating that there still was a weak force between thrombin and P10.

### FT-IR spectroscopy analysis

**Fig. 5:**
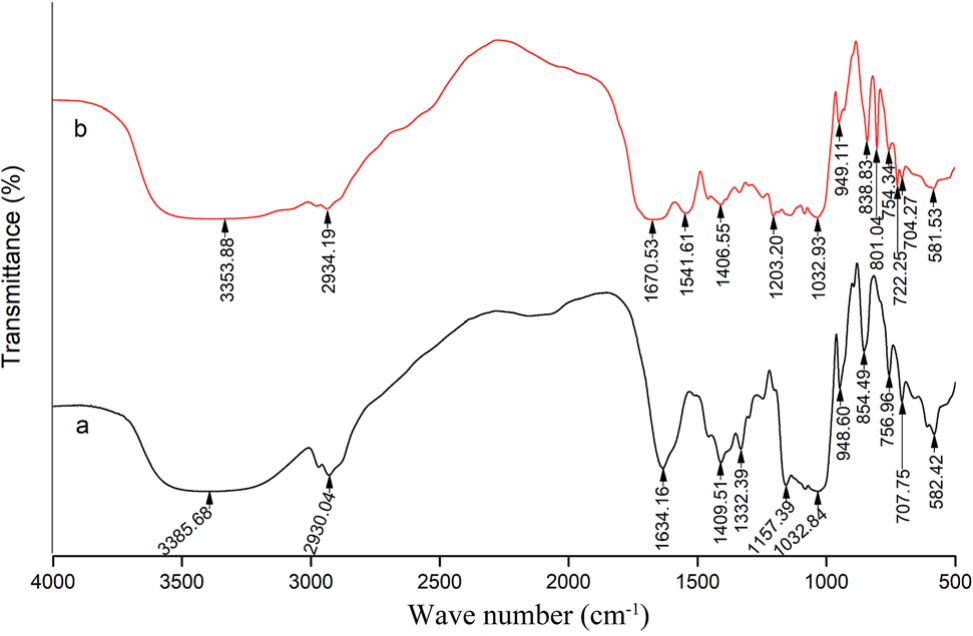
FT-IR spectra of thrombin (a) and thrombin plus 80 μM P10 (b). Both of the concentrations of thrombin were 5 μM.

Fig. 5 shows the characteristics of the thrombin infrared spectrum with and without the P10. There was no significant change in the main infrared absorption spectrum of thrombin with the presence of P10, but some characteristic peaks have changed.

The absorption peak of amide A usually appears at 3400 cm^−1^ to 3440 cm^−1^, and when it is associated with the hydrogen bond, it will shift to a lower wave number.^45^ It can be seen that the wave number of thrombin was from 3358.68 cm^−1^ to 3353.88 cm^−1^ with the addition of the P10. This was most likely due to the hydrogen bonding force between P10 and thrombin, which caused association between molecules, causing the absorption frequency of the amine group to move in the low wave direction. Moreover, it was in agreement with the previous fluorescence analysis that one of the forces between P10 and thrombin was hydrogen bonding.^45^ The asymmetric stretching vibration of the CH_2_ of amide B had a weak absorption at 2930.04 cm^−1^, and shifted the peak to 2934.19 cm^−1^ with the addition of P10. It indicated that P10 interfered with the asymmetric stretching vibration of CH_2_ of thrombin.

The peak displacement between 1600 cm^−1^ and 1700 cm^−1^ is the C=O stretching vibration of the protein polypeptide skeleton, which is a sensitive region for the change of the secondary structure of the protein.^46^ The typical absorption area of the protein secondary structure is identified as follows: 1646–1661 cm^−1^ is α-helix, 1615–1637 cm^−1^ and 1682–1698 cm^−1^ is β-sheet, 1661–1681 cm^−1^ is β-turn, and 1637–1645 cm^−1^ is random coil.^46^ The peak of thrombin at 1634.16 cm^−1^ was moved to the longer wave at 1670.53 cm^−1^ in the presence of P10. Obviously, this showed that P10 could destroy the β-sheet of thrombin, which was the same as the CD spectroscopy result.

The band between 1200–1360 cm^−1^ is amide III, which is caused by C–N stretching and N–H bending. Also, the CH_2_ rocking vibration peak of glycine and proline side chain is in this region.^47^ In thrombin, the characteristic frequency of the proline side chain CH_2_ swayed at 1332.39 cm^−1^. When P10 was added, the characteristic peak disappeared, indicating that P10 might interact with proline. In addition, the peak at 1157.39 cm^−1^ disappeared and the peak of 1203.32 cm^−1^ appeared, indicating that the C–O stretch was affected by P10.

When the wave number is less than 1000 cm^−1^, it is mainly C–H out-of-plane bending. The absorption peaks in this area are dense and complex.^48^ The wave number of 854.49 cm^−1^, 756.96 cm^−1^, 707.75 cm^−1^ and 582.42 cm^−1^ shifted to 838.83 cm^−1^, 754.34 cm^−1^, 704.27 cm^−1^ and 581.53 cm^−1^ by adding P10, respectively. These wave numbers had undergone short-wave displacement to varying degrees, indicating the change of the C–H microenvironment when the P10 interacted with thrombin.

### Raman spectroscopy

**Fig. 6:**
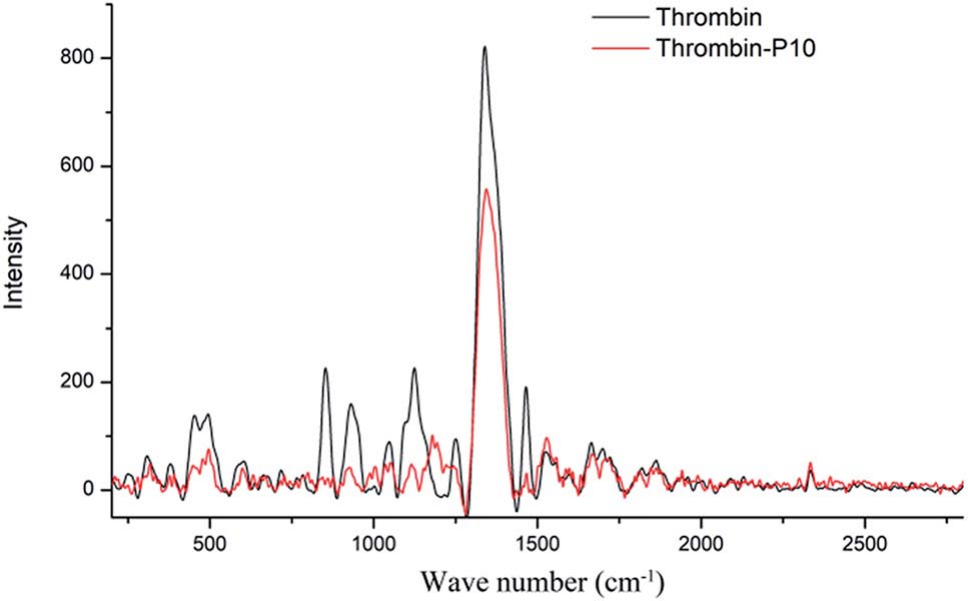
Raman spectra of thrombin and thrombin plus 80 μM P10. Both of the concentrations of thrombin were 5 μM.

Fig. 6 is the Raman spectra of thrombin and thrombin plus 80 μM P10. Raman spectroscopy mainly comes from the vibration of the molecular chain or side chain of the biomacromolecule. Similar to the infrared spectrum, however, the strong band generally appearing in the infrared spectrum becomes a weak band or even does not appear in the Raman spectrum. The secondary structure or conformational change are determined by analyzing the position and intensity of the amide bond.^49^

Thrombin had proline characteristic peaks at 450 cm^−1^, 494 cm^−1^, 853 cm^−1^ and 931 cm^−1^ (Fig. 6). After the addition of P10, the intensity of these characteristic peaks was significantly reduced without significant displacement. This clarified that P10 had only a weak interaction with proline and did not affect the microenvironment.^50^ 1048 cm^−1^ and 1124 cm^−1^ were NH_2_ rocking/twisting vibrations. After P10 interacted with thrombin, the characteristic absorption decreased at 1048 cm^−1^ and 1124 cm^−1^, but a new peak appeared at 1189 cm^−1^. The possible reason is that P10 binds to the side chain NH_2_ of the thrombin, which affects its characteristic absorption. The Raman at 1251 cm^−1^ was attributed to the amide III band, which is due to the peptide bond C–N stretching and N–H bending vibration, C_α_–C stretching and C=O in-plane bending.^51^ The decrease in the intensity of P10-thrombin in this band indicated a decrease in the degree of β-sheet folding of thrombin. This further validated the results of the previous CD and IR. In addition, the strong intensity absorption peak of thrombin at 1339 cm^−1^ was decreased in the presence of P10 and was slightly shifted to long waves (1346 cm^−1^). This indicated that P10 interacted with part of the thrombin side chain CH_2_ or CH_3_ and affected the surrounding microenvironment.^51^ The thrombin lysine side chain C–H vibration caused a characteristic peak at 1465 cm^−1^. And the intensity of the peak at this point was significantly reduced when the P10 was combined with lysine.^52^

## Experimental

### Materials

**Scheme 1:**
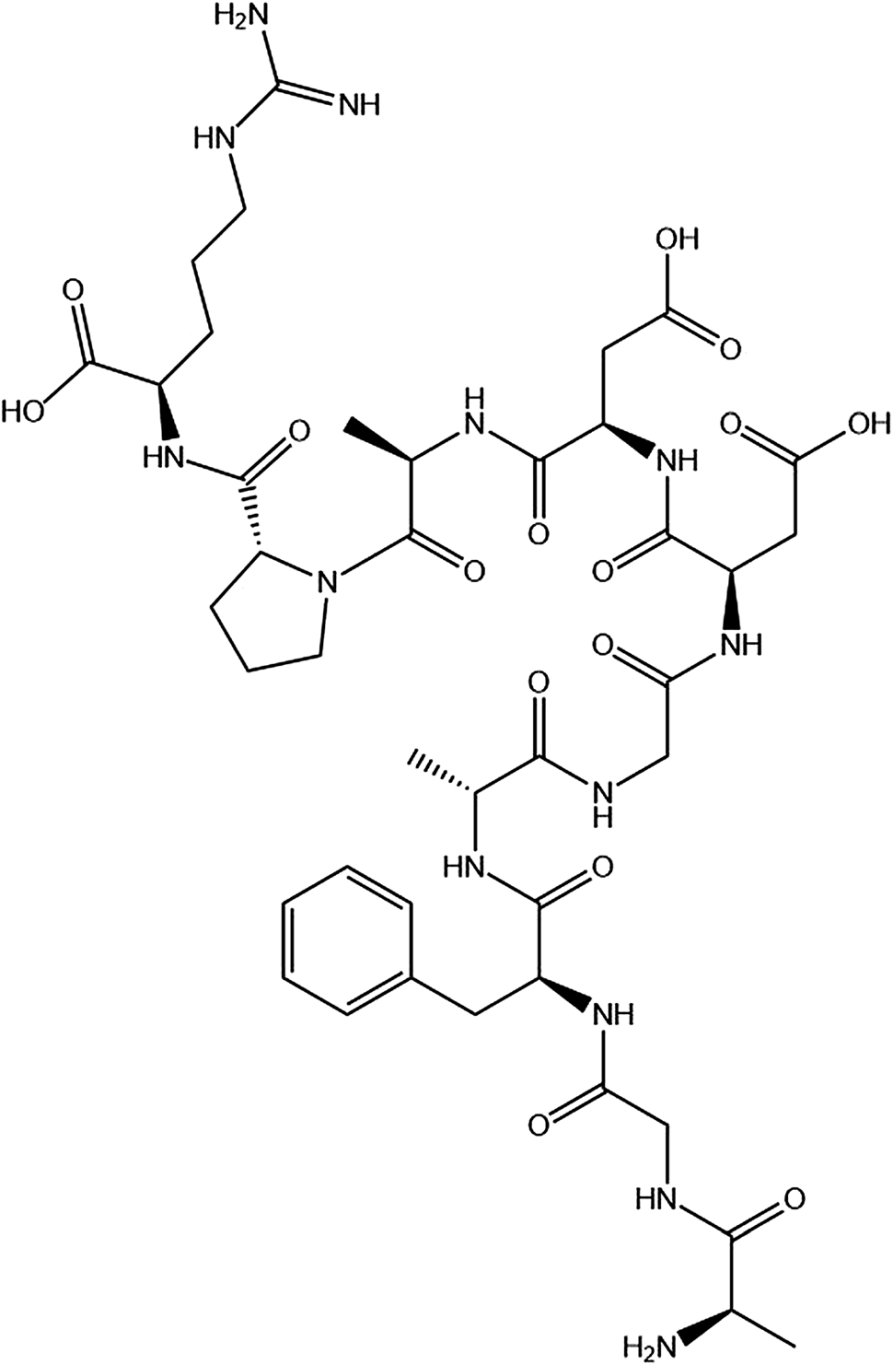
The chemical structure of the synthesized peptide P10 (AGFAGDDAPR). The image obtained by ChemDraw.

The P10 (Scheme 1) was synthesized by Shanghai Bootech Bioscience & Technology Co., Ltd. (Shanghai, China) with high purity (≥95%). Bovine fibrinogen and thrombin (40-300 NIH units per mg protein) were purchased from Sigma-Aldrich Co. (St. Louis, MO, USA). All the chemical reagents used were analytical grade unless indicated otherwise. The water used in this experiment was double distilled water.

### Determination of the antithrombotic activity

Antithrombotic activity was determined according to the method of Yang *et al.*^53^ The samples of thrombin and fibrinogen were dissolved in 0.05 M pH 7.4 Tris–HCl buffer, respectively. The determination was carried out using the IMark plate reader (Uji-shi, Kyoto, Japan) at 405 nm. 140 μL of 1 mg mL^−1^ fibrinogen and 40 μL of the sample were added to the microplate, then mixed and the absorbance was recorded as *S*_b_. After, 10 μL of thrombin (12 IU mL^−1^) was added to the mixture which was maintained at 37 °C for 10 min, and the absorbance was recorded as S. 40 μL of buffer instead of the sample was used for control group, and the absorbances were *C*_b_ and *C*, respectively. The antithrombotic activity was calculated from eqn (5) as follows:5
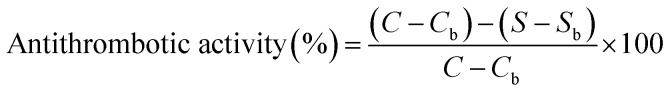


### Fluorescence spectrometry

The fluorescence spectra were recorded on the Hitachi F-4500 fluorescence spectrophotometer (Hitachi, Japan). 1 mL of different concentrations of P10 (0, 5 μM, 10 μM, 20 μM, 40 μM and 80 μM) and 2 mL of thrombin (5 μM) were mixed and incubated for 10 min at 310 K and 290 K, respectively. The fluorescence emission spectrum of the mixture from 290 nm to 420 nm was scanned, and the excitation wavelength was set to 280 nm. The excitation and emission slit width was set to 5 nm.

### UV-vis spectrometry

The UV-vis absorption spectroscopy was determined by a UV-2600 spectrophotometer (Shimadzu, Japan). The UV-vis absorption spectra of thrombin and the P10-thrombin mixtures were obtained at room temperature in the range of 185–400 nm. The concentrations of thrombin and peptide were 5 μM and 80 μM, respectively.^54,55^

### CD spectra

The CD spectra of the samples in the range of 185–250 nm were recorded on a JASCO J-810 automatic recording spectropolarimeter (Tokyo, Japan) controlled by the Jasco software with a 1 cm quartz cell at room temperature. In the samples, the concentration of thrombin was 5 μM, and the concentrations of P10 were 0, 10 μM, 40 μM and 80 μM, respectively. Each sample was scanned three times to find the average for a CD spectrum.

### FT-IR spectroscopy

FT-IR spectroscopy analysis was carried out using a Fourier-transform infrared spectroscopy analyzer (Thermo, Massachusetts, USA). Thrombin and thrombin plus 80 μM P10 were freeze-dried to obtain dry powders and separately finely ground with KBr to form transparent sheets. The scanning range was 500-4000 cm^−1^ and the resolution was 4 cm^−1^.

### Raman spectroscopy

The measurements were carried out using a QE-Pro spectrometer (Ocean Optics, USA) with a laser source wavelength of 785 nm and a power of 320 mW. The molar ratio of thrombin and P10 was 1 : 16 in the thrombin plus P10. The measurement range was 800–2870 cm^−1^ with scanning 2 times and the acquisition time was 25 s.

### Statistical analysis

The data was analyzed using IBM SPSS software 22.0 (IBM, Chicago, IL, USA). The chart data was smoothed and analyzed using OriginPro 8 SR4 software (OriginLab Co., Northampton, USA).

## Conclusions

Spectroscopic analysis is a simple and effective method to study the interaction between small molecules and proteins. All spectroscopy results indicated that P10 can interact effectively with thrombin. P10 was primarily bound by hydrogen bonding and van der Waals forces with thrombin in a 1 : 1 ratio. The binding of P10 to thrombin was an exothermic process and produced static fluorescence quenching. In the secondary structure, P10 reduced the β-sheet structure of thrombin and transformed it into random coils. P10 bound to proline and lysine of thrombin. Therefore, P10 changes the structure of thrombin, resulting in a decrease in its enzymatic activity.

However, in the fluorescence quenching experiments, time-resolved fluorescence studies (measurement of fluorescence lifetime) are more reliable than temperature-dependent studies to distinguish between static and dynamic quenching mechanisms that can be used to further explore the interaction of thrombin and modified P10. And we cannot visually obtain a model of P10 and thrombin binding. This requires an auxiliary computer to simulate molecular docking to obtain a docking model. In future studies, it is necessary to conduct *in vitro* or *in vivo* experiments to verify the effectiveness of P10 in antithrombotics and lay the foundation for clinical applications.

## Conflicts of interest

There are no conflicts to declare.

## Supplementary Material
